# Virtual reality for the observation of oncology models (VROOM): immersive analytics for oncology patient cohorts

**DOI:** 10.1038/s41598-022-15548-1

**Published:** 2022-07-05

**Authors:** Chng Wei Lau, Zhonglin Qu, Daniel Draper, Rosa Quan, Ali Braytee, Andrew Bluff, Dongmo Zhang, Andrew Johnston, Paul J. Kennedy, Simeon Simoff, Quang Vinh Nguyen, Daniel Catchpoole

**Affiliations:** 1 School of Computer, Data and Mathematical Sciences, Western Sydney University, Parramatta, Australia; 2 School of Psychology, Western Sydney University, Penrith, Australia; 3 Samurai Punk, Melbourne, Australia; 4 School of Computer Science, University of Technology Sydney, Ultimo, Australia; 5 Biospecimen Research Services, Children’s Cancer Research Unit, The Kids Research Institute, The Children’s Hospital at Westmead, Westmead, Australia; 6 MARCS Institute and School of Computer, Data and Mathematical Sciences, Western Sydney University, Parramatta, Australia; 7 Rollerchimp, Sydney, Australia

**Keywords:** Cancer, Computational biology and bioinformatics, Genetics, Biomarkers, Diseases, Health care

## Abstract

The significant advancement of inexpensive and portable virtual reality (VR) and augmented reality devices has re-energised the research in the immersive analytics field. The immersive environment is different from a traditional 2D display used to analyse 3D data as it provides a unified environment that supports immersion in a 3D scene, gestural interaction, haptic feedback and spatial audio. Genomic data analysis has been used in oncology to understand better the relationship between genetic profile, cancer type, and treatment option. This paper proposes a novel immersive analytics tool for cancer patient cohorts in a virtual reality environment, virtual reality to observe oncology data models. We utilise immersive technologies to analyse the gene expression and clinical data of a cohort of cancer patients. Various machine learning algorithms and visualisation methods have also been deployed in VR to enhance the data interrogation process. This is supported with established 2D visual analytics and graphical methods in bioinformatics, such as scatter plots, descriptive statistical information, linear regression, box plot and heatmap into our visualisation. Our approach allows the clinician to interrogate the information that is familiar and meaningful to them while providing them immersive analytics capabilities to make new discoveries toward personalised medicine.

## Introduction

The distinctive potential of visual and immersive analytics in pattern recognition of visualisation combined with state-of-the-art data analysis and machine learning techniques is critical to turn these data into insights and knowledge usable in the real world^1^. Visual analytics enhances the user understanding of the abstract nature of data in forming pattern recognition, insight, and relationship discovery data^2–6^.

Immersive analytics is an application of visual analytics that uses immersive methods to support the analysis, such as virtual reality and augmented reality visualisation^7,8^.

The recent and rapid growth of affordable low-cost, high performance and portable head-mounted display (HMD) like SONY PlayStation VR headset, Oculus Quest/Rift, HTC Vive, and Microsoft HoloLens, combined with the integration of feature-rich game engine platforms, such as Unity and Unreal Engine, have fostered the resurgence of immersive analytics research^9–11^. Increased accessibility to these game engine platforms free the researchers from dealing with low-level graphical manipulation, which is usually very labour intensive, to concentrate on developing graphical data mapping, natural user interface, and fluid navigation.

Moloney et al.^11^ have a useful discussion on the need for further research into immersive analytics by considering the affordance theory of the immersive technologies. The inclusion of aural, haptic, and kinaesthetic in the visualisation can benefit the analytics because the multisensory inputs are naturally encoded within human beings, and when used correctly, it improves the perception of the information and cognitive processing^11^. These are the distinct advantages over 3D visualisations on traditional 2D displays, such as desktops, laptops, and tablets.

The multi-dimensional nature of the genomic data combined with a patients' clinical profile and treatment history requires machine learning techniques to reduce it to manageable dimensions that facilitate visual analytics^12^. These machine learning algorithms primarily conduct knowledge extraction and predictive analytics of probable outcomes. The strength of visualisation is that it allows the human subject to visually discover patterns that are too complex or dynamic to be identified just by machine learning alone, especially in analysing the overview and details of the data and their relationship. Feature selection can be made manually by subject matter experts or used in conjunction with machine learning algorithms and subject matter experts to validate the features^13–15^. Feature selection and dimension reduction are used to allow the multi-dimensional data to be viewed in a three-dimensional environment we term a ‘similarity space’ where the relative position of each patient in the space is a measure of their similarity of selected features. For visualisation as similarity space, dimension reduction algorithms such as Principal Component Analysis (PCA) or Multi-dimensional scaling (MDS) are used with clustering methods to group patients with similar genetic profile^16^ which is followed by the application of visual analytics to explore and identify new relationships between individual patients directly within the patients’ cohort. Both Filho et al.^17^ and our study^18^ have successfully employed 3D immersive scatterplot in order to visualise dimensionally-reduced data more efficiently in their respective domains.

VR/AR visualisation being widely adopted in the medical field as summarised in Qu et al.^19^. Most of its adaptation is in diseases diagnostic, volume rendering and modelling Computed Tomography (CT) and Magnetic Resonance Imaging (MRI) data^20–24^, with immersive visualisation being offered as a treatment option in pain management^25,30^ and used extensively in patient rehabilitation^26–29^. In bioinformatics, immersive visualisation has also been used to investigate the correlation between genes and diseases^31^. Different from the above, our work focuses on patients’ cohort data interrogation in the VR/AR environment. VR/AR are used in medical domain mainly for human interaction support. There are few attempts to use VR/AR for interaction with data about patients and in clinical decision making.

In the postgenomic era, the vast amount of information generated from patient genetic profiles have made visualisation crucial in bioinformatics as it allows us to make sense of the data^1,32^. Genomics data analytics have been developed on traditional displays^33–40^, mobile devices^41,42^, large immersive environments^43,44^, and adopting Unity3D game technology to support the fluid and seamless analytics in the 3D environment (such as^18,45–48^). Techniques in this approach have been effectively applied in analysing genomic and cancer data as reviewed in Qu et al.^49^. Techniques for genomics data analytics in immersive environment are gaining traction. CellexalVR^50^ designed to deliver single-cell RNA sequencing data (RNASeq) data visualisation in an immersive virtual reality environment, aims to enable the immersive analytics of such data coupled with multiple statistical data analytics toolsets to take advantage of the unlimited space in the virtual space that is not available in the 2D display. MinOmics, which uses UnityMol to run on multiple platforms and manages the biological data from storage to analysis and visualisation of proteomic and transcriptomic data^48^. StarMap is an immersive visualisation for single-cell RNASeq data which, unlike CellexalVR^51^, focuses on the mobile web browser on a VR-enabled smartphone. Lastly, BioVR^52^ extends UnityMol as a VR-based platform for visualising DNA/RNA sequences and multi-omics data and protein structures in aggregate. BioVR currently runs on Oculus Rift and uses Leap motion for user interaction.

None of the above methods provide users with a visualisation to deep-dive into the genomic similarity with patient treatment history required for the clinical assessment of a single patient within the context of the wider cohort. Our work addresses this gap by integrating the similarity data with different clustering algorithms and treatment history for the clinician to guide their understanding of the patient’s condition.

This paper proposes a novel application of an immersive visual analytics framework with supporting visual design, environment optimisation, machine learning algorithms, and visual analytics that enables the discovery of patterns from known biomarkers and their relationship with patient cohort and history, as supported by a systematic study Qu et al.^19^. It provides an interactive and informative empirical-based system for data interrogation. The framework aims to provide oncologists and clinicians with a visualisation that helps them to drive their analysis in treatment efficacy and achieve the goal of evidence-based personalised medicine. The innovation in our work is the seamless integration of the latest discovery in cancer patients prognosis, the latest development in immersive technologies, machine learning, and interactive visualisation to allows a complete visual analytics platform of complex genomic data in a VR environment. The main contributions of this paper include:

- A design for an immersive visual analytics platform in a VR environment for end-to-end interrogation of patients’ data within a specific cohort on the basis of the similarity of the genomic changes that characterise their tumours, which also accounts for patient treatment histories and clinical indicators.
- A novel and effective visual design of a VR environment that is built on our usability study of exocentric and egocentric visualisations through optimisation of audio and visual design principles.
- An intuitive user interface and immersive visual analytics environment that implements similarity space for patient cohorts displayed in well-known 2D genomic charts and graphics with meaning to the biomedicine field, such as box plot, dendrogram and heatmap to ensure the visualisation is easily perceivable by clinicians and oncologists in their patient assessment.
- An optimisation of immersive assets including “real-world” mimicking virtual environment to provide a smooth user interaction experience and speed usability in the environment.
- A detailed walkthrough of the VR environment via a case study that illustrates the effectiveness of our work in a real-world application.

## Results and discussions

Our approach was to be in line with recent studies which showed that egocentric frame of references reduced the mental load needed while exocentric references led to better performance^53,54^. We proceeded our development with the combination of both exocentric and egocentric views to utilise the usability of the two designs.

### VROOM design specifics

**Figure 1:**
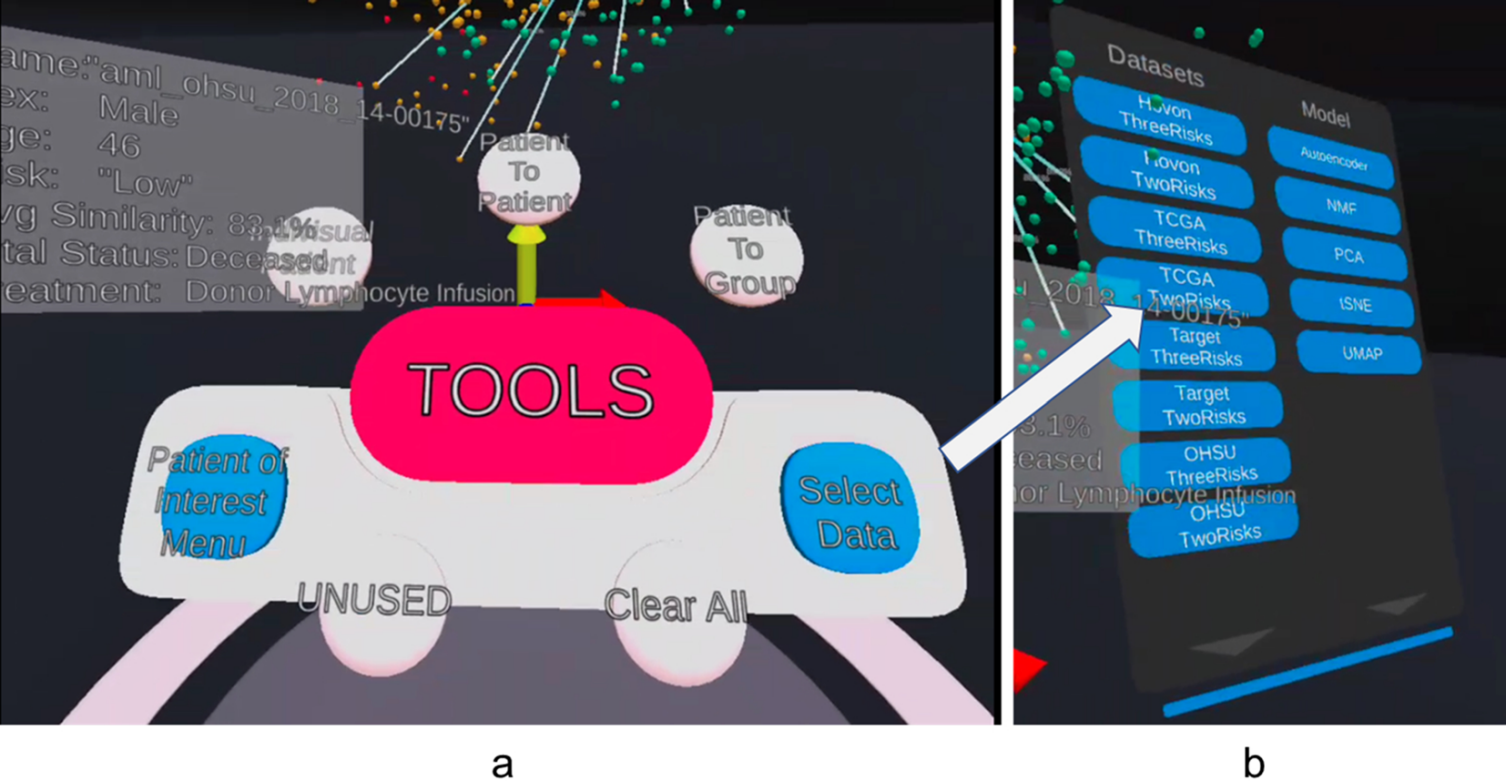
The Visualisation has four datasets rendered in similarity space based on five different dimensional reduction algorithms. (**a**) Our user interfaces with the options available for detailed analysis. (**b**) The four primary datasets categorised into two and three risk factors with Autoencoder, NMF, PCA, tSNE, and UMAP dimensional reduction algorithm.

The study's objective is to match a clinician's need to understand their patient of interest (POI) by interrogating the POI in the patients’ cohort in the similarity space. The process of interrogating a POI is a comparative exercise in which doctors will want to examine a group of patients similar to the POI with the expectation that this may allow the doctor to expect a common response to a set of treatments. The clinician's comparison mechanism changes from the patient presentation and blunt pathology test to more profound, more refined molecular information captured in their genes and genetic activity. Our visualisation allows the clinician to identify the patient(s) for comparison (POC). Once POI and POC (s) are identified, the clinician can conduct a detailed analysis of the patient in our visualisation. Currently, the POC(s) selection can either be the nearest ten neighbours in the similarity space or driven by the game theory as an alternative guided analysis. We aim to develop an analytical tool to guide the clinician in their prognosis and treatment regimen development by making these comparisons. Our design specifics will have following characteristics:

- The main visualisation is the 3D similarity space of the patients based on their genomic profile and been clustered based on their genomic similarity relied on the chosen dimensional reduction algorithm as shown in Fig. 1. We will go through the three use cases of the visualisation: (1) Patient details; (2) Patient to patient comparison; (3) Patient to group comparison. The result of the design study has been integrated into the immersive visualisation.
- The visualisation design incorporated tablet-like panels and 2D windows to display box plot, histogram, heatmap, hierarchy clustering dendrogram and tabular form. The ultimate goal of this work is to facilitate the discovery of new knowledge based on genomic similarity and possible application in personalised treatment.
- By working closely with the domain expert, the visual design had undergone an preliminary evaluation with a small number of cancer research experts and clinicians at the Children's Hospital at Westmead, Sydney, Australia^18^. The qualitative comment on the early prototype provided us constructive feedback on our work so that we could improve our current development. For example, the comment on enhancing the ability of highlighting specific and associated individual patients or patient group(s). This comment helped us to improve the visualisation with visual links between the selected patient and the nearest neighbours in the similarity space.
- To reduce oculomotor discomfort in the immersive environment, it is suggested^54,55^ that using virtual body and physical movement of body help in reducing such discomfort. In our implementation, we minimise the walking by placing the user interface and data visualisation in front of the user by placing the system in a dome-like environment on a platform.
- We fully utilised immersive multisensory capabilities to engage our users in the visualisation. With the low cost of these VR/AR devices, 3D design has become very common, and with urgent needs of working from home, 3D presence is being developed and tested for daily use. Our user interface mimics the real-world object as suggested by Pangilinan^56^ in the form of a virtual desk. The virtual desk concept has also been applied successfully by Wagner Filho et al.^57^. It creates an environment that the users can intuitively know how to use.
- When crafting our visualisation, the application of these design principles has been integrated and carefully orchestrated. Our design is simple to use and mimic real-world objects to ensure user familiarity. The use of audio feedback is beneficial when interacting in the VR environment to inform the user of the occurrence of the action.
- For immersive visualisation, proper sound design can create a sense of presence, especially in connection with the visual cue^58,59^, and it is essential in interaction^60^. Bleeker et al.^61^ describe data sonification as the sound rendering technique to provide access to data and in response to interaction events. Massiceti et al.^62^ show improvement in term of task completion time, velocity and other navigational behaviours with the help of sonification. Song and Beilharz^63^ argue that sonification should be both aesthetic and informative and presented evidence that data sonication improves user experience. However, when designing the sounds, care must be taken to avoid repetition and ensure fatigue avoidance, such as a simple option to allow users to turn both the background sound and action sound on or off^59^. An immersive environment soundscape may have two types of sounds: passive and active sounds^64,65^. Passive sound is the background sound or music to create a general atmosphere; while, active sound engages our awareness, usually in response to the event triggered by our action^58,59,64,65^.

Based on the sound design principles above, we have implemented three main sonic components in our study: data sonification, user interaction and ambient sound. With the lack of tactile feedback, audio becomes vital in providing interaction feedback to the user. Data Sonification aids the user when traversing a large data set by providing supplementary sonic feedback that is unique to each data point. Similarly, every action taken by the user will trigger a corresponding sound event to affirm the outcome of the action. Ambient sound is used to immerse the user and enhance their sense of presence in the virtual world. Initial sonification prototypes emphasised a high-fidelity correspondence with the underlying data points where possible, mapping sonic frequency, volume and wave shape directly to various data parameters. While it produced an accurate sonification, the large data sets created a highly cacophonous soundscape that was unpleasant to experience and difficult for users to comprehend.

To make the overall sound design pleasant and harmonious for the user, the final sound design for VROOM uses five guiding principles for each sonic component: aesthetically pleasing, congruent with action, congruent with visuals, distinctive signature and spatially informative. The ambient, user interaction and data sonification components all share the same pentatonic scale and musical key, but each separate component has unique instrumentation and resides in a separate frequency and spatial domain. This design allows the user to easily distinguish between any ambient, user interaction and data sonification sounds while maintaining a harmonious overall soundscape. While more pleasant for the user, this approach relegated the data sonification to merely supplement the visualisations rather than providing any new information on its own and this could be any area to readdress in future studies.

### VROOM case study: AML

Our visualisation currently is designed to visualise the genomic similarity data for Acute Myeloid Leukaemia (AML) patients with a 2D tablet-like toolset for detailed analysis of the patients. The visualisation adopts the exocentric view for the similarity space for the user to start their inspection of the data; and, if further details analysis is required, the user can either walk into the dataset or use zooming and panning to be inside of the data to take an egocentric view for details inspection. This has been proven to be effective by Risch et al.^66^.

**Figure 2:**
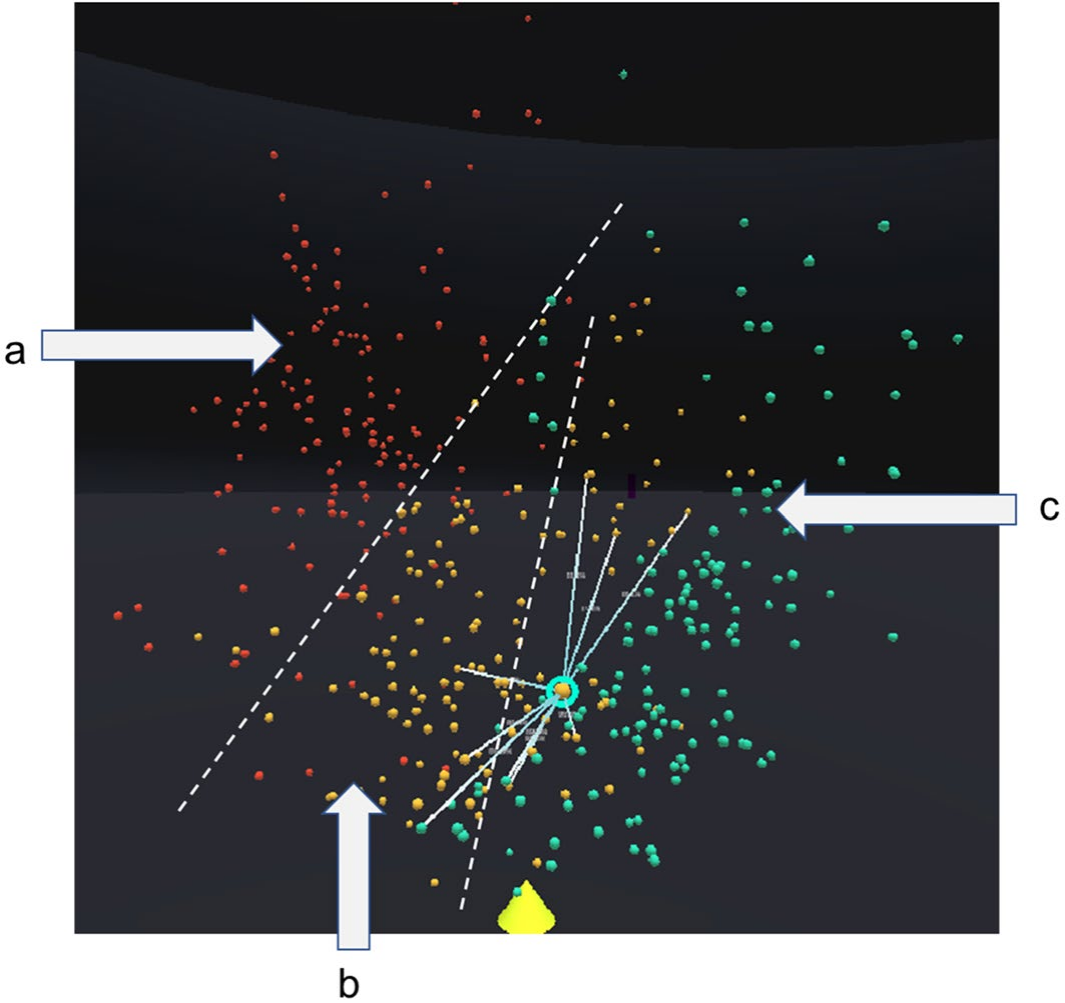
A close-up of the 3D similarity space of the patient cohort for the OHSC threerisk dataset. Colours are used to indicate the risk level. The cohort can roughly be divided into three regions, a, b and c.

In our Visualisation, the gene expression signature called leukaemia stem cells-17 (LSC17) analysis score^67^ is used for the list of gene-of-interest for AML. This is further clustered and dimensionally reduced into 3D space. The default dataset for the cohort space is "Hovon ThreeRisk" dataset, and the default dimensional reduction algorithm is autoencoder. For our walkthrough analysis, we will use "OHSC ThreeRisk" dataset and autoencoder as the choice of selection. The POI is "aml_ohsu_2018_16-01272". The POCs are “aml_ohsu_2018_16-01272” and “aml_ohsu_2018_16-00316”. The POCs for group comparison is based on the recommendation from the game theory. For visual identification, our POI is also the currently selected patient, which has a green halo over the sphere, and it is oversized compared to the rest of the sphere, as shown in Fig. 2.

Figure 2 shows the similarity space in the visualisation. The colour of the sphere (red: high risk, yellow: medium risk, and green: low risk) is used to represent the patient's risk level based on their LSC17 score. LSC17 score indicates the risk of a patient who might not respond well to standard therapy and should be considered for novel alternative treatment^67^. Figure 2 clearly shows that the patient cohort can be roughly divided into three zones labelled, a, b, and c. If a new patient introduced into the system falls into one of the regions, for example, region a, there is a high possibility that the patient has a high risk of rejecting the standard treatment. The details of the patient and the comparison of POI to POC and POCs can be done with the visual analytics tool. The clustering also allows the analysts to choose their POC if they want to look at similar patients or dissimilar patients by comparing patients in the same region or patients from the region a and region c to validate if their treatment regimen affects survivability.

**Figure 3:**
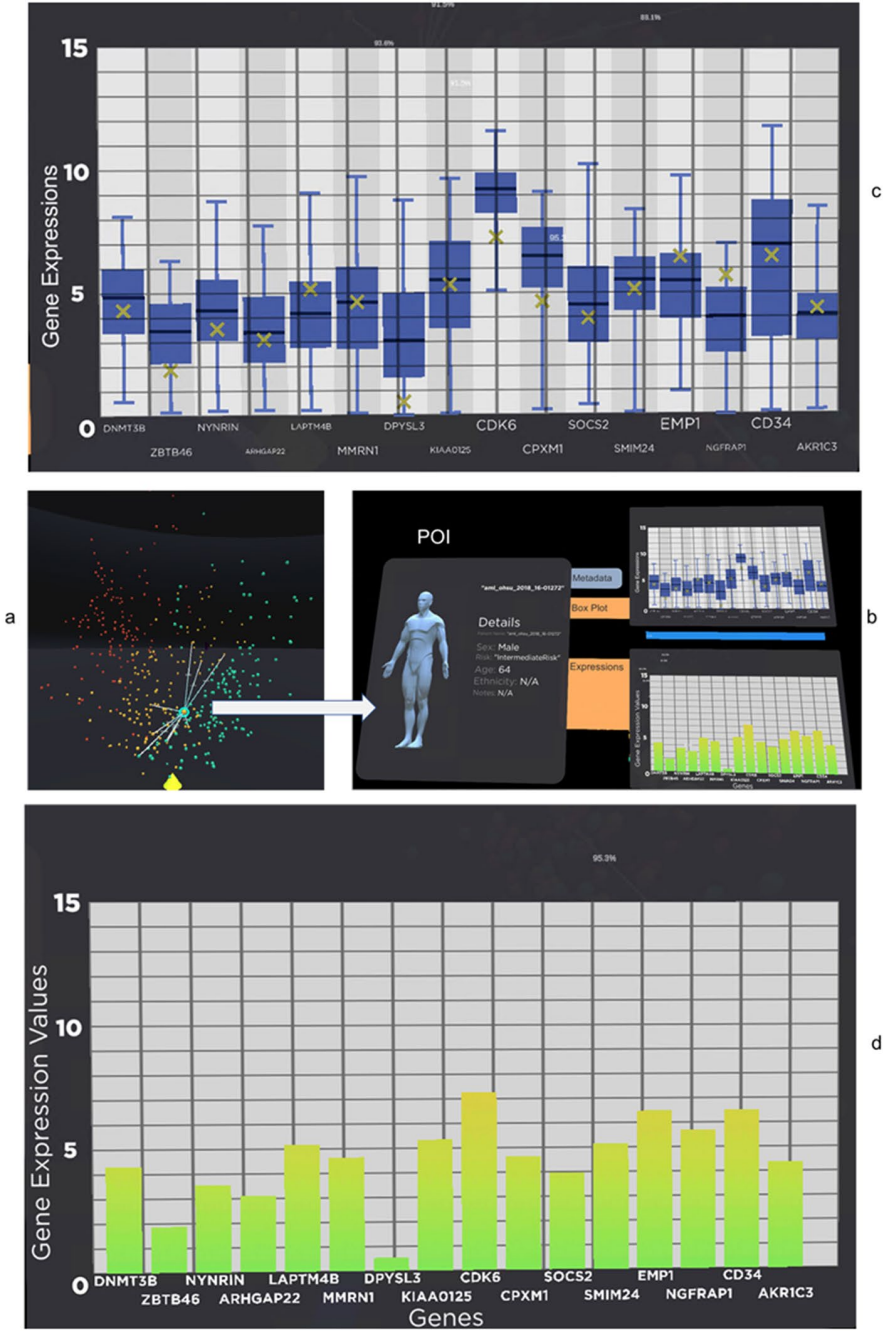
Patient "aml_ohsu_2018_16-01272" is selected from (**a**) and drag into (**b**) patient of Interest panel. The panel shows the metadata for the patient, such as age, gender and ethnicity. (**c**) The tablet also contains the box plot of the patient gene data compared to the whole population. The patient gene expression value is clearly marked as an "x" (yellow) in the box plot. (**d**) A bar chart of the gene expression value.

Once the analyst decides the patient to be investigated, in our case, "aml_ohsu_2018_16-01272", the analyst can drag the patient from the similarity space and collide it with the Patient of Interest Panel for detailed analysis as shown clearly in Fig. 3 with the use of the virtual hand in immersive space controlled by the Oculus Touch controller.

Figure 3 shows the type of visual analysis that can be applied to the POI. The panel is created to have a tablet look-and-feel to tap into the user's familiarity with the 2D tablet interface. The analyst can grab and move the panel around the immersive environment to organise and maximise their working space. Metadata for the patient is displayed on the left of the panel. In our case, patient "aml_ohsu_2018_16-01272" is a 64-year-old male whose LSC17 score risk is intermediate. The patient's gene expressions for the LSC17 gene list are displayed in box plot and bar charts on the right of the panel. Figure 3c is a box plot for comparing our POI "aml_ohsu_2018_16-01272" gene expression value to the rest of the cohort in the OHSC dataset. It is noticeable that most of the POI's gene expressions are within the interquartile range (IQR) of the box plot. Figure 3d shows the POI's gene expression in the form of a bar chart. An observation that can be made here is that although a gene can be highly expressed in terms of value, for example, CDK6, for our POI, it is not as activated as the rest of the cohort since it is below the Q1 in the cohort. According to GeneCard(R) The Human Gene Database^68^, "CDK6, along with its partner CDK4, are key players in cell cycle progression. The complex has been implicated in several cancer types and is the focus of therapeutic research and development. One targeted therapy for CDK inhibition is palbociclib, which may slow the growth of advanced-stage breast cancers. It has also been shown, in mice, that CDK inhibition may sensitise mutant PIK3CA tumours to PI3K inhibitors".

This type of in-depth analysis can help the analyst better understand the patient prognosis and potential risk and treatment choices, giving them the ability to drill down to gene-level expression.

#### Patient-to-patient comparison

**Figure 4:**
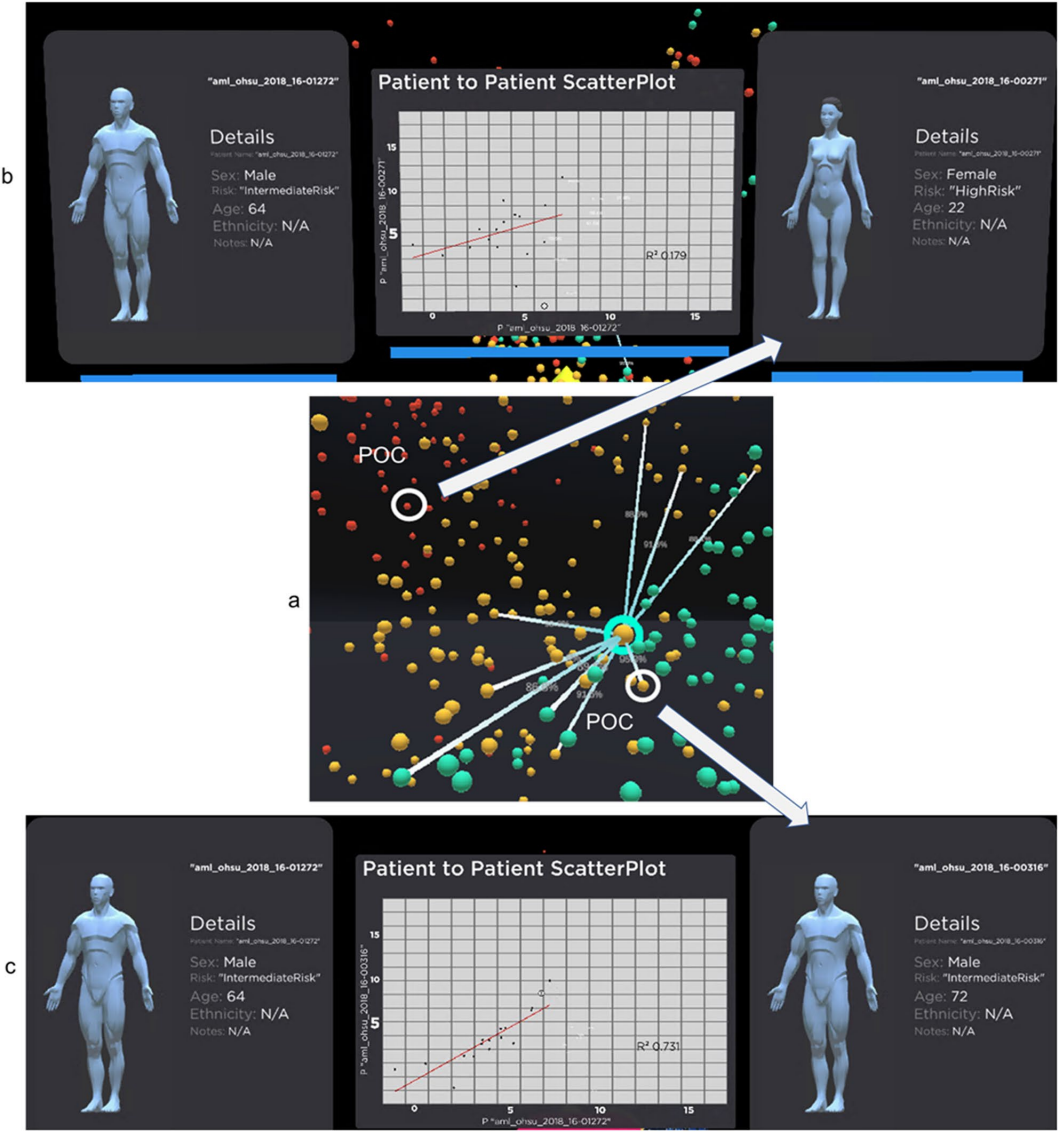
Patient-to-Patient Comparison panel. (**a**) The patient “aml_ohsu_2018_16-01272” is our POI, circled with green, while "aml_ohsu_2018_16-00271" and “aml_ohsu_2018_16-00316” are our POCs, circled with white. (**b**) POC—"aml_ohsu_2018_16-00271” is used to show the dissimilarity of the gene expression from POI. The scatter plot is used to analyse the similarity between the patient in term of their gene expression. These two patients have a similarity rate of 85%. When regression was carried out on the data, the model does not fit well, with only 0.179 R-square value. (**c**) In contrast, “aml_ohsu_2018_16-00316″ is very similar to POI at 91.5%. Regression model fit well with R-square value of 0.731.

Our visualisation allows the analysts to compare the POI to a POC. This would allow the analyst to understand how similar the patient is by using linear regression. Figure 4 shows the process of comparing our POI to two POCs from different clusters in the 3D cohort similarity space. When the analyst's virtual hand hovers over the sphere, representing patient n, tooltips will provide an overall gene similarity between the selected patient and highlighted patient n (blue). This will give the analyst a sense of the similarity for the patient they are choosing for comparison.

We will continue to use the same POI, "aml_ohsu_2018_16-01272", for the following 2 case studies. A POC, "aml_ohsu_2018_16-00271", from a cluster further away from POI, is chosen in our first case study. This is a scenario where the analyst wants to see how dissimilar the POC is from the POI, Figure 4b shows the metadata for our POC, a 22-year-old female with a high-risk factor for LSC17 score. As shown in Figure 4b, the regression line does not fit well due to the dissimilarity of the two patients. The R-square value is 0.179. R-square is the squared multiple correlation or coefficient of determination for our linear regression model. The closer the R-square value to 1, the better the linear regression is describing the data. It is also called the goodness-of-fit measure for our regression model^69^.

For our second case study, the POC, "aml_ohsu_2018_16-00316", is chosen who is roughly located in the same cluster as the POI, Figure 4c shows the metadata for this POC, a 72-year-old male with an LSC17 risk factor of Intermediate. This POC has an overall similarity with POI at 91.5%, which is higher than the POC in the first case study. The R-square for our linear regression model, at 0.731, also confirm the better degree of goodness-of-fit of the data.

The scatter plot can also provide a visual cue of how many genes are very different for the POI and POC. If the two patients have the same gene expression profile, the linear regression should have a y=x equation.

#### Patient-to-group comparison

Our key novel innovation is the patient-to-group comparison panel. It is implemented similarly as a tablet. The Patient-to-Group panel is the most feature-rich. The patient-to-group analysis would require the analyst to select a POI and a group of POCs. The POI selection is made using the Oculus Touch button. The system can help select two distinct groups of POCs, or the analyst can pick any POCs of their choice. In the first case, the system can identify the nearest nine neighbours with the highest overall similarity to the POI. Our decision support system engine drives the second type of POC selection. The POCs are visually linked to the POI using white colour lines. The automation of the analysis is used to help identify which combination of three genes out of the LSC17 combo will yield the highest survival rate when analysed separately.

**Figure 5:**
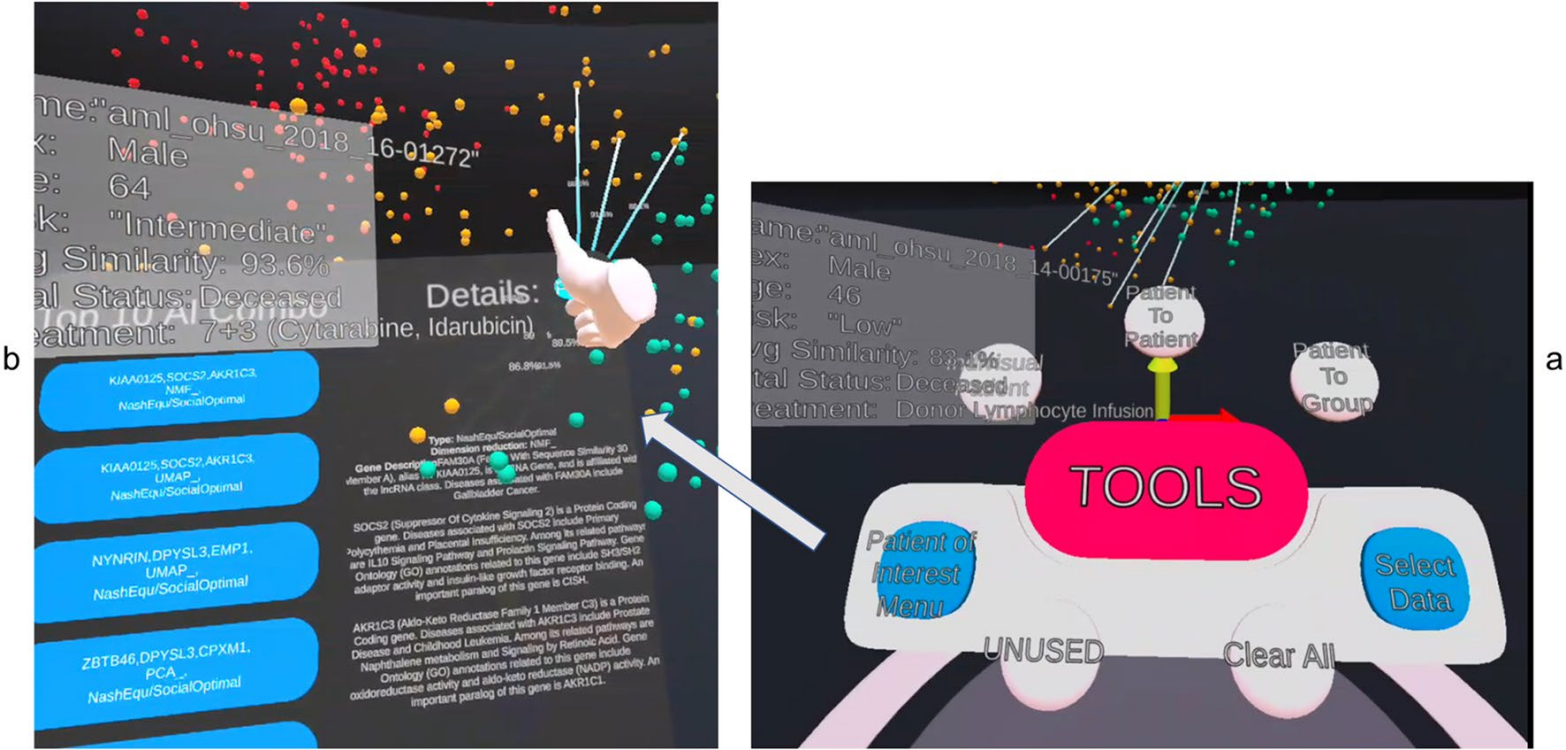
In the 3D similarity space, our POI is highlighted and linked by straight lines to some other patients as suggested by our AI. (**a**) To use the Decision Support System., the analyst can push the "Patient Of Interest Menu" button. The list of top 5 combos will be listed on the left side of the screen as shown in (**b**). The details of the gene description extracted from GeneCards® are displayed on the right. This is one of the Overview and details analysis keys to visual analytics that can be conducted within this similarity space. By selecting different combinations, the neighbours linked by the straight line will change if that neighbour is no longer part of the relationship. This is particularly useful if the gene combination has a special meaning. The selected groups of POCs can be then sent to Patient-to-Group Panel for further analysis. In this case, the selected combination is “KIAA0125”, “SOC2” and “AKR1C3”, which was found using NMF, and the result was a Nash Equilibrium and social optimal solution for game theory.

Figure 5 shows the flow on how to use the decision support system engine to assist in patient analysis. The Decision Support System can be triggered by clicking on the "Patient of Interest menu" (Fig. 5a). A panel will be shown with the top 5 combinations of the gene, dimension reduction algorithm and if the combination is a result of Nash equilibrium or social optimal result on the left of the panel. The right panel will have the genes' description in question extracted from GeneCards®(Fig. 5b). By selecting the different combinations, the highlighted neighbours would be changed accordingly.

This is one of our novel implementations of Overview & details complex analysis which involved looking at the overview of the data while conducting a separate and independent drill-down analysis of the data to allow complex immersive analytics to be conducted. The analysts can then go back to the similarity space to inspect the newly identified POCs suggested by the Decision Support System. To complete the POCs selection for our patient-to-group panel, the analyst can send this data to the panel using the P2G button. For our case study, the selected combination is the top item, which has the gene combination of KIAA0125, SOC2 and AKR1C3, which was found using NMF, and the result was a Nash Equilibrium and social optimal solution for game theory.

**Figure 6:**
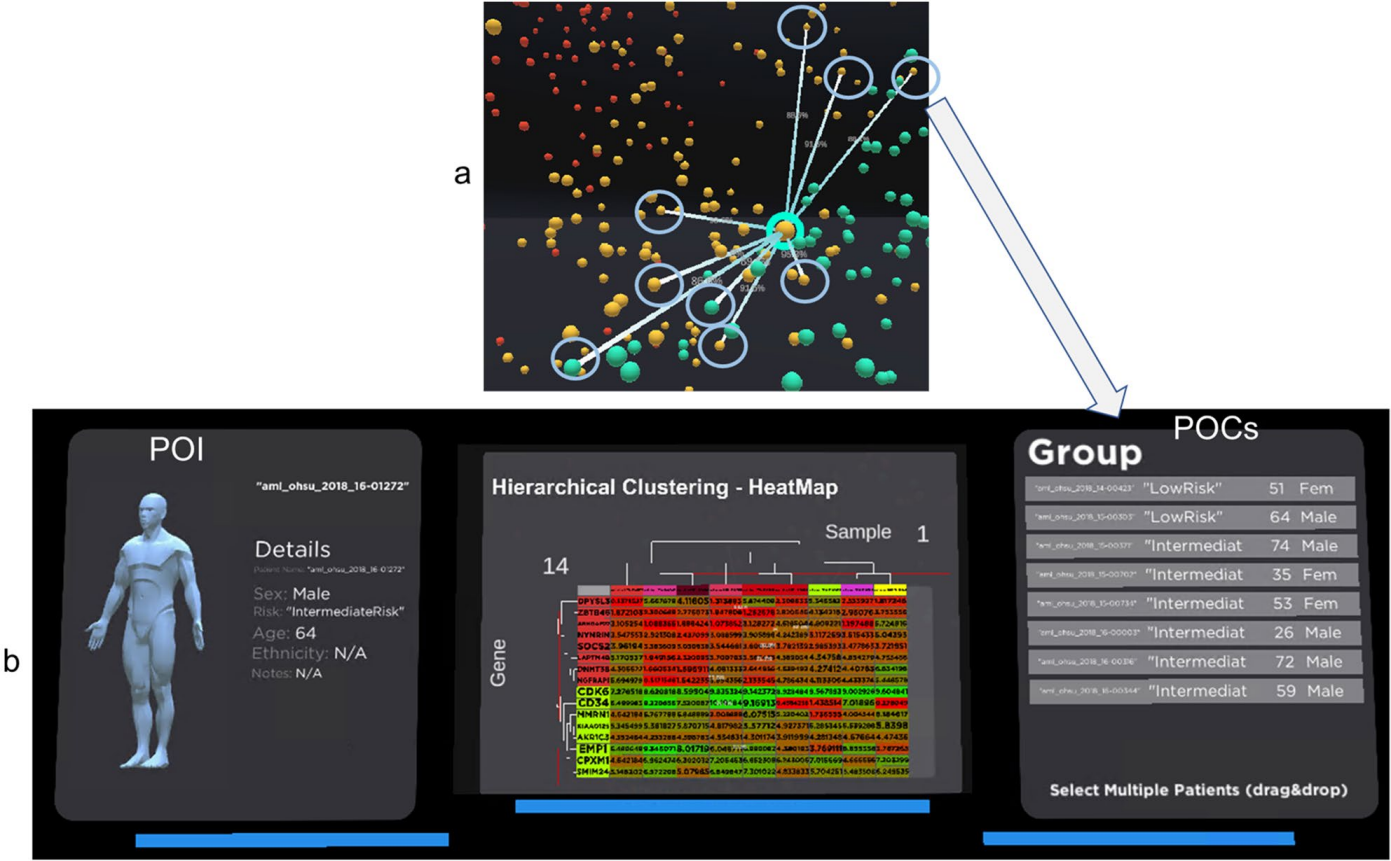
Patient-to-Group comparison panel. The analyst using the decision support system to trigger the selection of POI and POCs in this panel. The primary patient is our POI as shown in (**a**). All POCs are highlighted in light blue in (**a**) and are listed under the group panel in (**b**).

**Figure 7:**
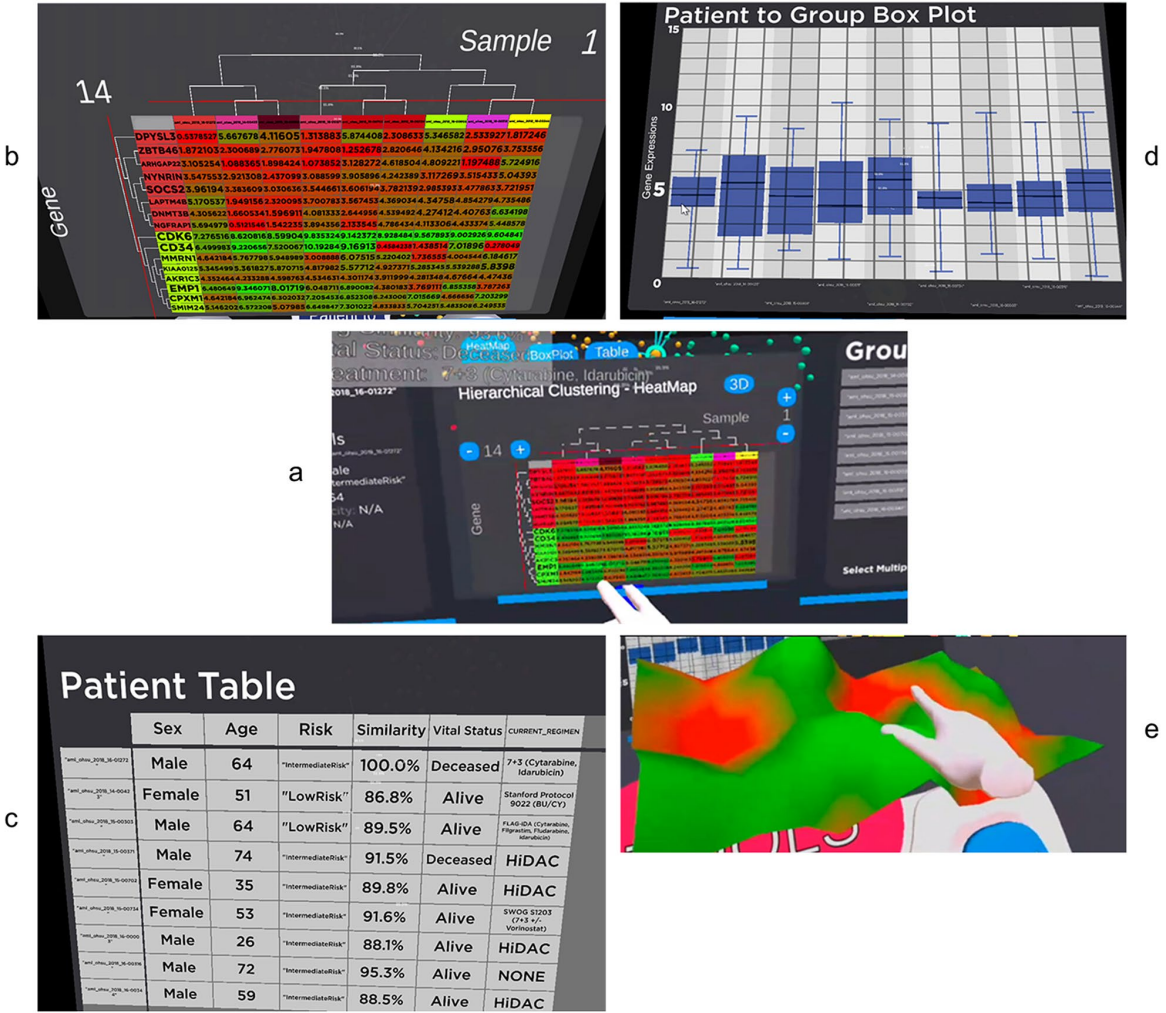
(**a**) The Patient-to-Group comparison panel has four visual analytics tools, hierarchical clustering heatmap, box plot, patient table and 3D heatmap. (**b**) The gene expression of the POI and the POCs are visualised in the heatmap. (**c**) Patients clinical history in tabular format. (**d**) The Box plot comparison of POI to POCs. The box plot shows the gene's median and IQR for the POI and the POCS. (**e**) 3D visualisation of the heatmap with the gene expression value determines the height of the mountain and the valley.

The patient-to-group panel will then be activated and be auto-populated with the POI and the POCs, as shown in Fig. 6. The patient-to-group panel will present the metadata for POI and the list of POCs on the right. Figure 6a shows the relationship and linkage between the POI and POCs. The POCs are highlighted with light blue circles. The patient-to-group panel (Fig. 7a) contains 4 visual analytics tool to drive the analysis: HeatMap (Fig. 7b), Patient table (Fig. 7c), Box Plot (Fig. 7d) and a 3D HeatMap (Fig. 7e).

**Figure 8:**
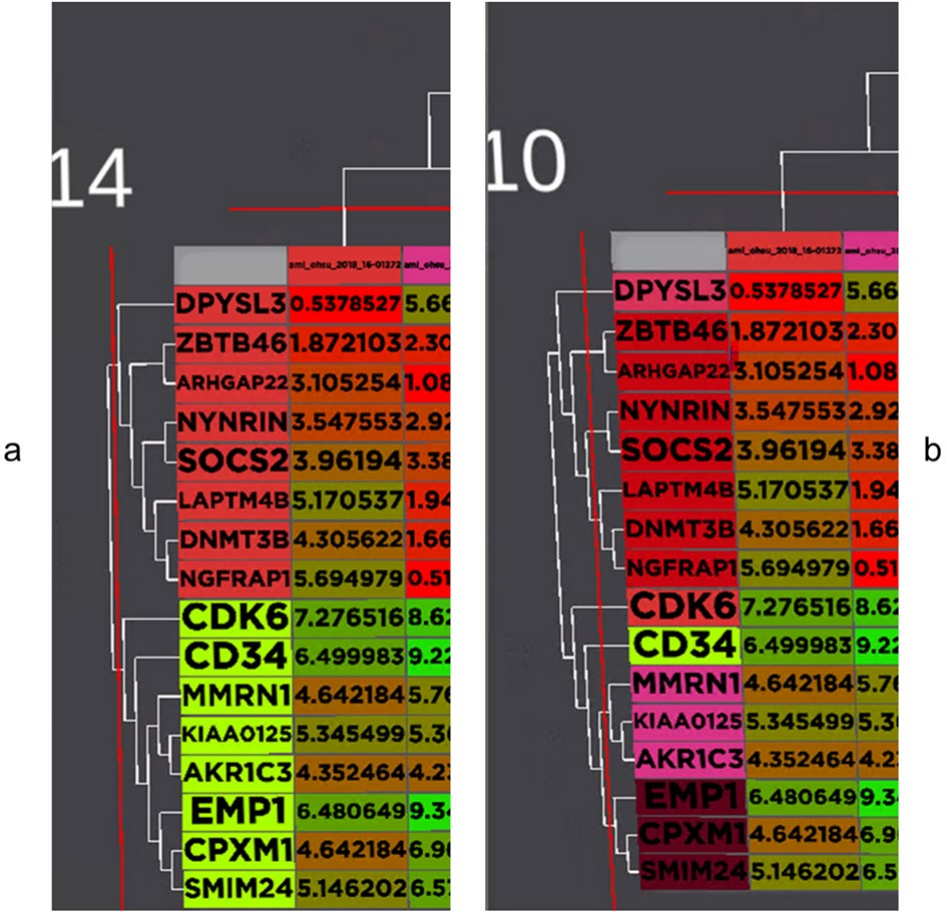
The heatmap is integrated with the hierarchy clustering and dendrogram. Red line is used to indicate the current level of clustering for both gene and sample. (**a**) The gene-level clustering was almost at the top of the tree. This created two clusters for the gene expression, as seen by the two different colours, in the row headers. (**b**) The level for gene clustering was lower to 10, which is about the middle of the tree hierarchy. At this stage, there are eight distinct clusters can be found for the gene. A similar study can be done for the sample hierarchy. Heatmap and dendrogram are very popular for gene expression visualisation.

Heatmap is very commonly used in genomic data analysis. It is a fundamental tool in visualising gene expression data to unravel hidden patterns in genomic data^49^. Our visualisation implemented the heatmap with the integration of the dendrogram using a hierarchical clustering algorithm (Fig. 8). Colours are used to indicate the level of expression for the gene (Red: low expression value, Green: high expression value). The level of hierarchical clustering of the heatmap can be changed with the + and − button. Changing the gene level from 14 to 10 will show a different clustering effect on the gene, as shown in Fig. 8. At level 14, the genes were clustered into two separate groups, while at level 10, the gene was clustered into eight distinct groups as shown in Fig. 8.

Box plot is available to analyse the POI and POCs (Fig. 7d). However, the implementation in patient-to-Group is different from those found in the patient-details panel. In this implementation, the box plots visualise the individual POCs and POI gene expression values. This will give the analyst an idea of the overall gene expression for these patients.

The patients' clinical history is available in tabular format (Fig. 7c). Most of the public dataset does not come with treatment and treatment history, but our OHSC dataset contains the current treatment regimen, which will be very useful for personalised therapy analysis after conducting an end-to-end analysis.

Lastly, a 3D visualisation of the heatmap is available for analysis (Fig. 7e). The visualisation was rendered with the gene expression value to determine the height of the mountain and the valley. It can be accessed with the 3D button. We intend to expand our future work to allow the analyst to walk on the 3D heatmap to experiment with the effectiveness of the future immersive capability of our visualisation.

#### Walkthrough of end-to-end analysis

**Figure 9:**
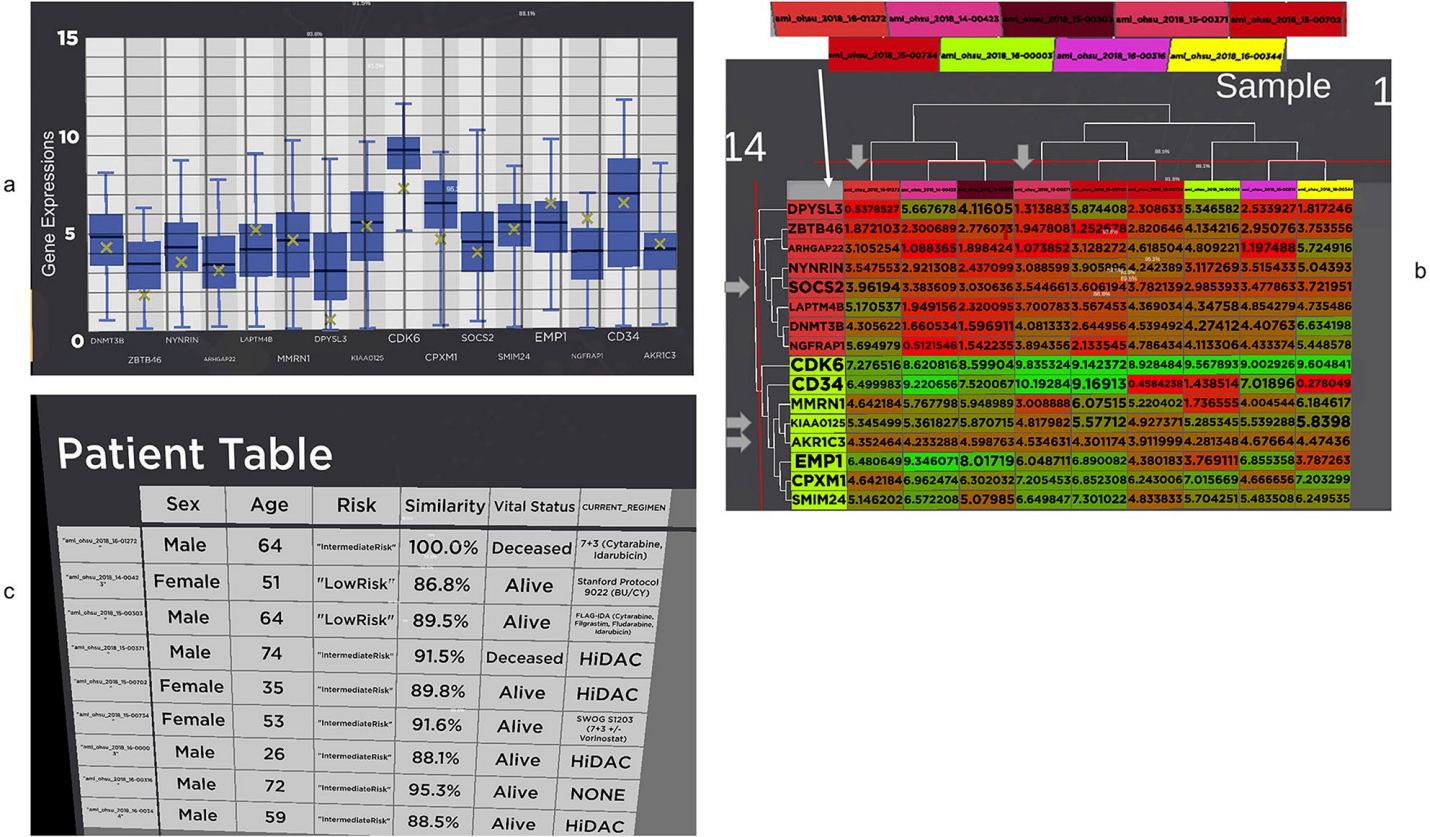
A walkthrough of an end-to-end analysis using the patient details panel and patient-to-group panel for overview and details analysis to unleash the knowledge embedded deep in the data for our POI and POCs. (**a**) First, the analyst can investigate the gene-of-interest, “CDK6”, expression value for the POI to see if it falls in the IQR range or an outliner. The analyst then can use the POCs suggested by the Decision Support System to investigate why these POCs cohort mostly survived the illness. (**b**) The analyst can then inspect the gene expression value for the POI compared to the POCs and conduct a hierarchical clustering analysis on the heatmap. Since the grouping was based on “KIAA0125”, “SOC2” and “AKR1C3” gene combination, the analyst can visually compare the differential expression for these three genes for POI and POCs. Since the similarity was done on these three genes, it made sense that those values shown in (**b**) were close to each other. (**c**) The medical history for all the patients. In this scenario, the analyst would find the POI and patient "aml_ohsu_2018_15-00371" were dead; and compare the regimen used for these patients. The visual comparison reveals that the regimen used for our POI is not the same as any of the rest of the POCs.

In this section, we will do a walkthrough of an end-to-end analysis using our immersive visualisation components such as 3D similarity space, the patient details panel and patient-to-group panel for overview and details analysis to discover the knowledge embedded deep in the data for our POI and POCs (Fig. 9).

We will continue to use the same POI and POCs identified earlier. First, using the patient-details analysis panel, the analyst can investigate the gene-of-interest, for example, CDK6, expression value for the POI, to see if it falls in the IQR range or an outliner (Fig. 9a). The analyst can choose their POCs or allow the decision support system to suggest a POCs group to analyse. In our case study, our analyst wants to understand how to find those nearest patients that are very genetically similar to the POI and have survived the illness. This is what our decision support system was designed to handle.

Once the selection for POCs is made, the analyst can conduct a thorough genetic analysis study using the heatmap with the integration of hierarchical clustering. This will allow them to unravel any hidden patterns in the data (Fig. 9b). The heatmap shown in B was based on the POCs suggested by the decision support system, which was based on the “KIAA0125”, “SOC2” and “AKR1C3” gene combination, as it is clearly shown in the heatmap that for those items (indicated by the white arrows), the colours are very similar across all the patients. The analyst can then concentrate their gene expression differential analysis on the rest of the genes.

After a thorough heatmap analysis, the analyst can check the medical history of all the patients (Fig. 9c). Taking a quick look at the table, the analyst will find that only two patients from the cohort passed away, our POI and patient "aml_ohsu_2018_15-00371". The last regimen given to our POI was not the same as the rest of the POCs. This might give our analyst an idea of what was prescribed to POI and how to form a hypothesis to be tested biologically. It is also noted that patient "aml_ohsu_2018_15-00371" was given the same regimen, "HiDAC", which is similar to the other three patients, but the patient did not survive. However, patient "aml_ohsu_2018_15-00371" is significantly older than those three patients. The former was 74-year-old, while the other three falls under the age of 60s. According to Cancer Institute NSW^70^, the treatment efficacy "for patients aged 60 years and younger the probability of survival after four years was 35% in the 100 mg/m^2^ group (CI 95% 27–43%), 40% for the 400 mg/m^2^ group (CI 95% 32–49%) and 52% in the 3 g/m^2^ group (CI 95% 44–60%) (*p* = 0.02)" and the side-effect for "high dose cytarabine chemotherapy was much more poorly tolerated compared to the lower doses in the above 60 years of age group". There might be a possible linkage that is worth further investigation by the analyst. The analyst can also go back to the heatmap to visually inspect if there are significant differences in the four patients treated with "HiDAC" to look for a pattern.

**Figure 10:**
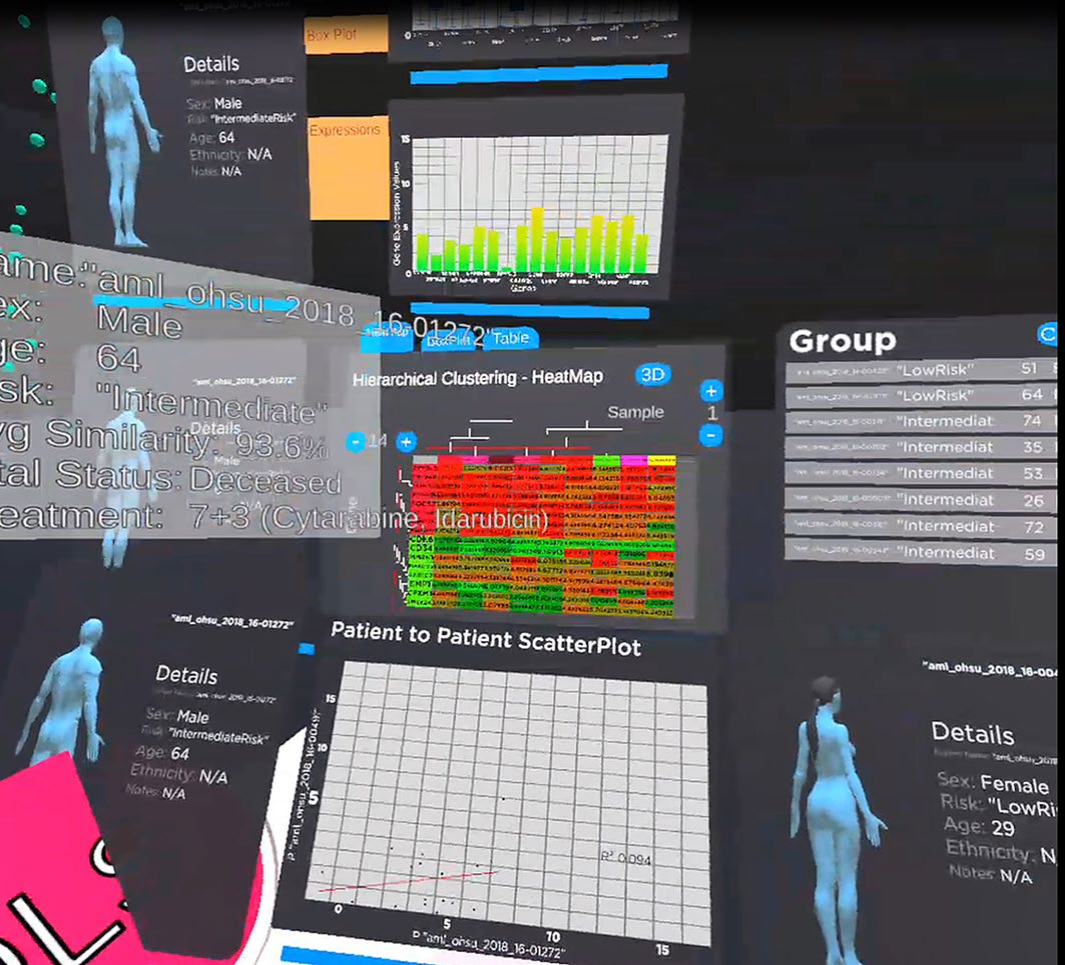
The analyst can stack all the panels anywhere in the immersive space to allow them to conduct a detailed analysis. This is possible in immersive space as there is unlimited space that can be used.

Lastly, the strength of our immersive visualisation lies in the fact that there is unlimited working space to allow the analyst to conduct in-depth and complex analysis without leaving the system. As shown clearly in Fig. 10, the analyst can stack the panel vertically on top of each other to have a clear visual inspection across the panels and, in comparison, to the 3D similarity space.

### User qualitative assessment

We have sought user assessment of our prototype with twenty participants (10 females and 10 males) representing clinicians (20%), genetic experts (40%), bioinformatic experts (20%), and health-related research students (20%). The greater number of potential participants were identified and invited by emails across two universities and a hospital in Sydney, Australia. Most of the participants (75%) had no or little experience with any VR device prior to the study. Each participant was instructed to complete a set of tasks based on a clinical scenario using our VROOM tool to assess the ease of usability, accessibility and interpretability of our prototype. For examples, the study asks the participants to interact and manipulate the visualisation to create comfortable views, select patients of interest to provide necessary visual analysis. The analysis includes examining the patients’ details as well as carry out comparisons for patient-to-patient and patient-to-group via graphical charts. A post-session questionnaire and verbal debriefing were carried to collect the participants’ feedback on the usability and the acceptability of the visualisation in the VR environment.

Results from our assessment showed that the participants highly regard the potential usefulness of the VROOM tool for distinguishing patient to patient comparisons based on the modelling of their genetic and biomedical information (10% very useful, 55% useful and 25% moderately useful). Specifically, 75% of the clinicians indicated that VROOM would be very useful for the patient analysis after their experiment with the clinical scenario. Qualitative comments showed that the participants expressed excitement that our VROOM VR environment allowed them to walk within the data, press a button, and select a patient for detailed assessment.

Regarding to the visualisation in the VR environment, we received very positive feedback from the participants on the accessibility of the current design in navigating the environment. Most of the responses indicated that the visualisation had an impact on the user perception and user experience (moderate impact 35%, impact 30%, and extremely impact 15%). We received constructive and positive qualitative feedback from the participants on the graphical design of the avatars where they helped them identify quickly additional information about the patients, such as their gender. Critical comments were also received on the design, such as the use of the red-green colour scheme not being so user friendly for colour deficient users and the need to showing additional information on the avatars, such as Body Mass Index (BMI), risk, and age. These limitations will be addressed in the next improvement of our VROOM prototype.

Most of the participants indicated that they noticed the sound during the trials which as instructive to them when completing their tasks (moderate helpful 15%, helpful 45%, and very helpful 20%). Qualitative feedback from the participants demonstrated that a “ding” sound which was congruent with action were helpful to bring to their attention that they have done a task correctly. The background music added positive experience in VR and helped them sustain engagement in the task.

We also identified that participants navigation in the VR space allowed them to investigate and find the information they need. 45% of the participants indicate that VROOM is “easy” to “very easy” to navigate and find the necessary information without any support, 25% of the participants could easily navigate with some help from the instructor, and 30% of the participants found it initially quite hard to navigate. This outcome reflects that our navigational design is in the right direction to meet the goal of the prototype, but there is a need for further improvement to a more intuitive navigation. In addition, sufficient training and practice are crucial to users who are particularly not familiar with VR and its navigational controller.

## Methods

### Design methodology: immersive analytics framework

This study complied with all relevant local guidelines and regulations. Our research only used data openly available through the public domain as listed in “Pre-Observation” section.

**Figure 11:**
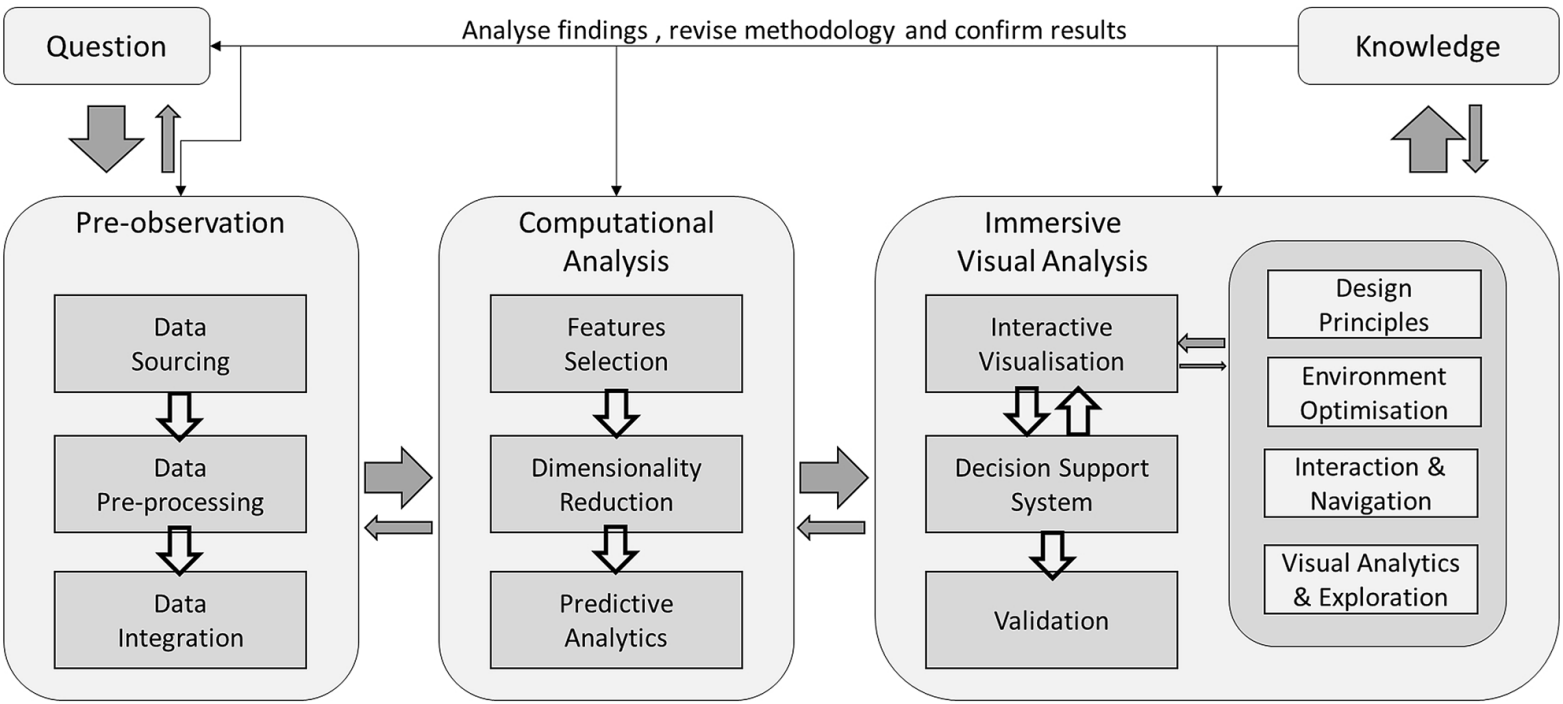
Our immersive visual analytics of genomic data for cancer patients extends Nguyen et al.^33^.

Our immersive analytics framework, which is an extension of our earlier work^33^ consists of multiple components that reflect a complete analytic cycle by integrating the immersive technologies and introducing game theory to power the Decision Support System (Fig. 11). Our earlier work^33^ on the framework has demonstrated its effectiveness in transforming complex genomic data into actionable and meaningful insight.

Efforts have been put into ensuring a proper, natural and optimised environment and asset design in an immersive environment following the well-established VR design principles. The components of the immersive analytics framework are briefly presented as follows.

Our system was developed using Unity3D software designed to run on Oculus Quest, Oculus Rift and Sony Playstation VR standard configuration. There is no other specific configuration required for the installation.

#### Pre-observation

Data sourcing includes the finding of the dataset to be used for the visualisation. The main key attributes in the data set are the tumour gene expression and clinical attributes.

For this study we used four different sources of the raw data used in this study in the form of bulk Ribonucleic acid (RNA) sequencing data, patient attributes and patient history. The data sources are from the TCGA Research Network (https://www.cancer.gov/tcga), HOVON (http://www.hovon.nl/), National Cancer Institute Office of Cancer Genomic—Target dataset (https://ocg.cancer.gov/programs/target/data-matrix) and dataset consolidated and used by^71^ (https://www.cbioportal.org/study/summary?id=aml_ohsu_2018). All the dataset are in public domain.

The patient attributes and clinical data is linked back to their RNASeq data to allow a thorough analysis of the data. These four independent data sources have been used and visualised in their own cohort.

Data pre-processing is essential when dealing with multiple sources of data representing the same gene. Typically, in bioinformatics, the analysis is performed on the differential gene expression. The magnitude of the gene expressed must be consistent across the dataset for the finding to be valid. Data from different sequencing technology, such as microarray or RNASeq, will be treated differently as two groups.

Data integration is the step where all these data are combined into a larger dataset. They are two types of integration, linking the patients' clinical history to their genomic profile; and, combining data from different sources into a large dataset. This is critical in genomic studies as features are always overwhelmingly larger than the sample size.

Multiple research findings on AML and biomarkers have been integrated into the analysis. The purpose of our visualisation is to enable clinicians to make an informed and empirical decision based on the consolidation and comparison of the various recommendations. The data are pre-processed with the input from medical experts in Acute Myeloid Leukaemia (AML) with the LSC17 score discussed earlier.

The main objective of our research is to do gene similarity analysis for the patients' cohort. The data is then processed with the computation analysis steps in our framework and made available in immersive space, as shown in Fig. 11.

#### Computational analysis

The purpose of visualisation is to derive actionable knowledge from the data. Our visualisation source the public domain data from multiple sources. All the data was in the form of RNASeq. RNASeq measures the average activity level for each gene, or gene ‘expression’, in a tissue or cellular specimen. Gene expression values are commonly investigated by cancer specialists to determine the activity of biological mechanisms ongoing in the tumour to explain pathological conditions^72,73^.

Feature selection can be conducted manually using studies that have proven to identify the gene associated with particular cancer. In our experiments, the data are pre-processed with the input from medical experts in Acute Myeloid Leukaemia (AML) with the gene expression signature called LSC17 score. It allows the analytics to be evidence-based with solid medical reasoning behind selecting the right genes for analysis^67^.

Dimensionality reduction, in this context, is different from feature selection. In data science, these terms are interchangeable to reduce the number of the features to train the model. We use dimensionality reduction to transform, cluster and project our patients into three-dimensional space for visualisation. We applied the latest dimensionality reduction built for visualisation such as t-distributed stochastic neighbour embedding (tSNE)^74^ and Uniform manifold approximation and projection (UMAP)^75,76^ and also long-established and tested algorithm such as Principal Component Analysis (PCA)^77–79^, Non-Negative Matrix Factorisation (NMF)^80^ and neural network-based auto-encoder^81,82^. By applying and allowing multiple algorithms to be used, we returned the power of choice to the user on what matters most to their analysis. For example, tSNE has a very strong local relationship while the global distance between dissimilar objects has no definite meaning beyond the fact that they are in different clusters; while, the PCA algorithm is heavily based on the variance in the data.

Predictive analytics is delivered using a machine learning server via web services to provide our tools with real-time machine learning and predictive capabilities. Currently, predictive capabilities are integrated in the dimensional reduction process to get the relevant features for our dataset. The predictive analytics model is currently under development, and future work will incorporate the predictive capability into the system. The idea is to allow the import of a new patient into the cohort and provide a survivability prediction based on the data available.

#### Immersive visual analysis

In our earlier work^18,33^, we have developed new visual analysis techniques that worked on 2D, 3D^33^, desktop space^33^ and an early immersive prototype^18^. In this paper, we will continue and extend our work into the immersive space.

Interactive visualisation is developed to provide an end-to-end analysis of the patient-of-interest (POI) cancer genomic profile compared with the genetically similar patients in the hope of finding evidence of commonality in treatment and treatment's efficacy. The visualisation provides an overview of the cancer patients' data cohort in similarity space and details analytical toolset that allows the drill-down into the detail. The experience is supplemented by the use of decision theory as the Decision Support engine to help drive the analysis and maximise the finding that would lead to the best outcome for the patient. To that end, our immersive visualisations include: (1) multiple three-dimensional visualisation of the patient cohort in the similarity space from four cohorts from public domain data; (2) Similarity space definition is driven by five-dimensional reduction algorithm with their strength in helping the discovery of knowledge; (3) Detailed patient information analysis box plot and bar chart; (4) POI to another patient comparison scatter plot with regression; (5) POI to another group of patients details analysis selectable by analysts with box plot of gene expression value, heatmap with dendrogram analysis and tabular format of patient attributes and medical histories such as current regimen and historical treatment information; (6) A 3D heatmap analysis of the similar heatmap found in (5).

Design principles are essential to ensure the usability of the visualisation. Our design follows LaViola^83^ guidelines to ensure simplicity, well-structured presentation, and visibility. Simplicity meant to keep the UI as simple as possible and make the system task focused. Structure dictated that UI should be organised in a meaningful and useful way. Visibility means the UI control should be self-explanatory and straightforward to use. Multisensory interfaces in the immersive environment, which includes sounds and haptics, is also be used to enhance the user experience^83,84^.

Environment optimisation is crucial in the VR environment to ensure a high-quality immersive experience for the user. Pangilinan^56^ stressed the importance of VR asset optimisation to ensure a solid natural visual experience. VR contents have to be developed to run at 90 frames per second (FPS) to ensure minimal discomfort to the user. A balance between a high poly count for the assets and VR rendering must be achieved by reducing the models’ poly count without sacrificing the asset shape or by minimising the texture size without reducing quality^56^. Sounds can play an essential role in enhancing the experience and providing information about the user's virtual environment^58,59^. The field of view in an immersive environment is around 100 degrees to 120 degrees, and an optimised sound design could reinforce the power of the visual and provide helpful information about the backdrop of the virtual environment^58,59,85^.

Interaction and navigation in an immersive environment can be categorised into four categories, navigation, selection, manipulation and system control^84,86^. These actions are enabled with the use of Oculus Touch. Navigation allows the user to explore the visualisation. Users can then select the POI for further investigation and use manipulation to mark the user for later examination. System control can be achieved through the various User Interface (UI) components, for example, patient to patient comparison panel.

Visual analytics and exploration is achieved by enabling immersive analytics. Immersive analytics allows visual analytics to be applied in an immersive environment. Immersive Analytics is achieved by integrating immersive technologies with natural user interface design to achieve a transparent cognitive experience environment^7,8,87^. Research has shown the effectiveness of immersive analytics to improve user engagement and productivity. We have examined multiple research projects to ensure we kept ourselves up-to-date with the designs and the best practices.

Decision support system is essential in VROOM to assist, but not overtake, the process of analysing the data. Game theory studies the interactions, particularly negotiation and coordination, between self-interested agents to maximise their returns in the form of utility value particularly^88–92^. Game theory has developed notions such as Nash equilibrium, Pareto optimal and social optimal for analysing such systems^88^. It is used in VROOM to help to drive decision making between clinicians that have differing opinions.

Validation is the last stage of the framework. It usually involves the domain expert to validate and confirm the results if the knowledge and hypothesis generated by the visualisation are acceptable and valid. User performance is analysed and measured based on the user’s feedback. It also helps to identify any gaps and potential design improvement of the visualisation in terms of user interaction.

### Ethical approval

The protocol of this study was reviewed and approved by the Western Sydney University Human Research Ethics committee and The Sydney Children's Hospital Network Ethics committee.

## Conclusions and future work

In this paper, our visualisation platform provides a novel immersive analytic framework to analyse and explore complex genomic and biomedical data. Visual and sound design principles and environment optimisation were carefully studied and applied to VROOM to ensure the immersive environment is natural and the user interface is fluid and intuitive. We apply domain users’ familiar visual charts and graphics in the biology field, such as heatmap, box plot, and dendrogram, in the immersive space that allow the domain experts to carry out their analysis without the burden of cognitive overhead. The walkthrough of the system in the case study demonstrates how clinicians can use the system to conduct their prognosis and how hypotheses can be formed for further investigation.

The purpose of the visualisation is to provide a mechanism for clinicians to go into the complex data visualisation and look at it from different angles, which is possible, especially with exocentric and egocentric views. That would allow them to see more specific inter-relationship of the data while engaging the data more naturally and physically in immersive space. The discovery of such relationships would be able to help them in forming hypotheses for future confirmation. A VROOM pilot user qualitative assessment was conducted with domain users to evaluate the platform’s effectiveness and users’ satisfaction in helping the clinician and analyst analyse patient information and treatment studies with some positive results on our design principle and use case.

## Supplementary Information

Supplementary Legends.

Supplementary Video 1.

## Data Availability

All the data used in this paper is in public domain as stated in the main text.
