# Supplementary material for: Virtual reality for the observation of oncology models (VROOM): immersive analytics for oncology patient cohorts
Source: Sci Rep. 2022 Jul 5;12:11337. doi: 10.1038/s41598-022-15548-1 (PMC9256599; doi:10.1038/s41598-022-15548-1)
Supplement: Supplementary file 1 — Supplementary Legends. [file 41598_2022_15548_MOESM1_ESM.docx]

An overview of the VROOM system, its features and design principles.  First, the visual design principle of the immersive system is discussed. VROOM sounds design is explained next, followed by user interaction and navigation system.  A walkthrough of the system usage is shared in detail as well.
